# AgBr/BiOBr Nano-Heterostructure-Decorated Polyacrylonitrile Nanofibers: A Recyclable High-Performance Photocatalyst for Dye Degradation under Visible-Light Irradiation

**DOI:** 10.3390/polym11101718

**Published:** 2019-10-19

**Authors:** Mingyi Zhang, Ying Qi, Zhenyi Zhang

**Affiliations:** 1 Key Laboratory for Photonic and Electronic Bandgap Materials, Ministry of Education, School of Physics and Electronic Engineering, Harbin Normal University, Harbin 150025, China; Zhangmingyi@hrbnu.edu.cn (M.Z.); qy1324759070@163.com (Y.Q.); 2 Key Laboratory of New Energy and Rare Earth Resource Utilization of State Ethnic Affairs Commission, Key Laboratory of Photosensitive Materials & Devices of Liaoning Province, School of Physics and Materials Engineering, Dalian Minzu University, 18 Liaohe West Road, Dalian 116600, China; 3 School of Materials Science and Engineering, Zhengzhou University, Zhengzhou 450001, China

**Keywords:** polyacrylonitrile nanofibers, flexibility, photocatalysis, interfacial charge–transfer, adsorption

## Abstract

Macrostructural flexible photocatalysts have been proven to have desirable recyclable properties during the photocatalytic degradation of organic pollutants in water. However, the photocatalytic activities of these photocatalysts are often unsatisfactory due to the fast recombination of charge carriers and the limited surface active sites. Herein, we developed a novel flexible photocatalyst of AgBr/BiOBr/polyacrylonitrile (PAN) composite mats (CMs) through the controllable assembly of AgBr/BiOBr nano-heterostructures on electrospun polyacrylonitrile nanofibers (PAN NFs) via a three-step synthesis route. The component ratio of AgBr to BiOBr in the CMs could be easily adjusted by controlling the in situ ion exchange process. The charge–transfer process occurring at the interface of the AgBr/BiOBr nano-heterostructures strongly hindered the recombination of photoinduced electron–hole pairs, thereby effectively enhancing the photocatalytic activity of the AgBr/BiOBr/PAN CMs. Meanwhile, the unique hierarchical inorganic/organic heterostructure of the AgBr/BiOBr/PAN CMs not only led to good flexibility, but also provided an abundance of active sites for photocatalytic reactions. Upon visible-light irradiation, AgBr/BiOBr/PAN CMs with an optimal ratio of AgBr to BiOBr components exhibited both enhanced photocatalytic activity and excellent separability during the degradation of methyl orange in water compared to the BiOBr/PAN CMs.

## 1. Introduction

With the rapid development of global industry and economics, there is an urgent demand for a “green” ecological environment for human beings. However, the heavy use of nonsustainable fossil fuels and industrial chemicals inevitably leads to environmental pollution threatening human health. [1,2,3,4]. Over the past several decades, many efforts have been devoted to environmental remediation using different physical or chemical techniques [5,6]. Among them, the “green” photocatalysis technique has attracted much interest, because it offers great potential for the degradation of organic pollutions in water or air by using high-performance semiconductor photocatalysts under sunlight irradiation [7,8,9]. As an indirect transition bandgap semiconductor, a BiOBr nanostructure with a low bandgap energy is considered to be an effective photocatalyst for visible-light-driven photocatalytic reactions [10]. The layered crystal structure of BiOBr provides a space large enough to polarize the electron–hole pairs, which can effectively boost the separation probability of photoinduced charge carriers during the photocatalytic degradation of organic pollution [11]. It has been reported that two-dimensional (2D) BiOBr nanosheets with good visible-light photocatalytic degradation activity could be easily obtained via a traditional hydrothermal process in the presence of cetyltrimethylammonium bromide (CTAB) as both the precursor and structure template [12]. Nevertheless, it is of immediate significance to further enhance photocatalytic activity and the recyclable properties of BiOBr nanostructures.

The rational integration of two different semiconductor photocatalysts to form a well-defined heterojunction interface has been regarded as an effective strategy in reducing charge recombination and enhancing photocatalytic activity. [13] According to band theory, the energy band structures of AgBr and BiOBr are suitable for the formation of “type II” heterojunctions in their heterostructures, which boosts the separation of photoinduced charge carriers during the photocatalytic process [11,12]. Although the AgBr/BiOBr heterostructure has been demonstrated to show enhanced visible-light photocatalytic activity for pollution degradation, the suspended particulate photocatalyst of the AgBr/BiOBr heterostructure is hard to separate completely in solution after a photocatalytic reaction, leading to repollution of the treated water. Thus, the design and fabrication of a macrostructural photocatalyst with a hierarchical multi-heterostructure seems to be an available method to solve the drawbacks to an AgBr/BiOBr heterostructure.

Immobilizing photocatalysts on macrostructural supports has been a common solution to answer the repollution problem of particulate photocatalysts. In various support materials, the electrospun mats of polyacrylonitrile nanofibers (PAN NFs) are promising for photocatalyst loading due to the following advantages [14,15]: (1) polymer PAN possesses a relatively stable molecular structure due to the existence of large numbers of cyano groups; (2) a nanosized fibrous structure with an ultralarge surface-to-volume ratio provides abundant active sites for the controllable immobilization of semiconductor-based photocatalysts; (3) electrospun NFs with random orientations interweave to form macroscopic flexible mats with micropore structures, achieving an easily tailorable photocatalyst; and (4) electrospun NFs with unique 1D nanostructure properties can realize a high level of exposure for the loaded nanostructure photocatalysts, therefore improving photocatalytic efficiency. To date, there has been no report on the fabrication of inorganic/organic composite photocatalysts for such AgBr/BiOBr heterostructure-decorated PAN NFs for visible-light-driven photocatalytic degradation.

In this work, we report on the controllable immobilization of AgBr/BiOBr nanostructures on the flexible support of electrospun PAN NFs through a facile solvothermal process combined with an in situ ion exchange method. The optimal AgBr/BiOBr/PAN composite mats (CMs) possessed enhanced photocatalytic activity and an excellent separability for the degradation of methyl orange under visible-light irradiation. This could be attributed to the effective process of charge–transfer on the AgBr/BiOBr heterojunction interface and the unique structural properties of flexible PAN nanofibrous mats. Our work provides new insight into the development of semiconductor heterojunction-based flexible photocatalysts for practical applications.

## 2. Experiment

**Scheme 1:**
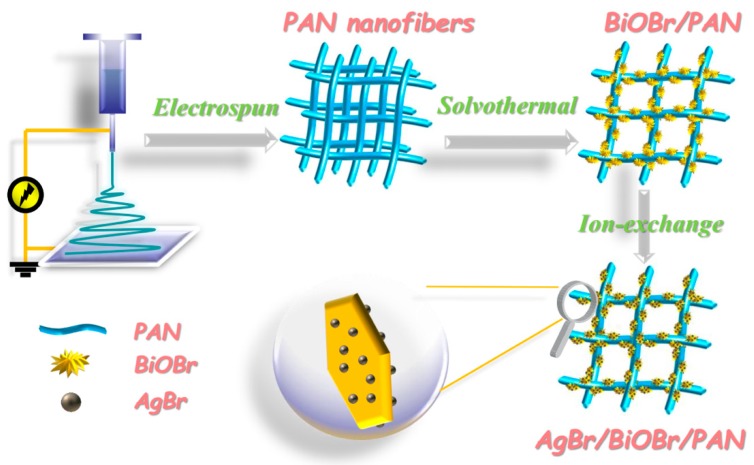
The schematic synthesis route of the AgBr/BiOBr/polyacrylonitrile (PAN) composite mats (CMs).

Scheme 1 illustrates the synthesis procedure of the flexible inorganic/organic composite mats (CMs) with different component ratios of AgBr/BiOBr nano-heterostructures on PAN NFs. First, the mats of the PAN NFs were obtained through an electrospinning technique. Second, BiOBr nanostructures were assembled onto the surfaces of the electrospun PAN NFs via a solvothermal treatment. Finally, a traditional ion exchange process was employed to construct the AgBr/BiOBr nano-heterostructures on the electrospun PAN NFs. During this process, the BiOBr could react with Ag^+^ ions in situ to form AgBr nanoparticles (NPs) in the presence of an ethylene glycol solution as the reductant:

$$\mathrm{BiOBr}+{\mathrm{Ag}}^{+}\overset{\mathrm{Room}\;\mathrm{Temperature}}{\to }{\mathrm{BiO}}^{+}+\mathrm{AgBr}↓.$$

Thus, AgBr/BiOBr/PAN CMs were obtained by using a three-step synthetic route.

### 2.1. Fabrication of PAN Nanofiber (NF) Mats

In a typical procedure, 1.0 g of PAN (Mw ca. 150,000, Sigma-Aldrich, Shanghai, China) powder was dissolved in 10 mL of *N,N*-dimethylformamide (DMF, Sinopharm Chemical ReagentCo., Ltd., Shanghai, China) solution. After vigorous stirring at room temperature for 12 h, a homogeneous solution formed. The above precursor solution was drawn into a needle tube. The working voltage was set at 7.5 kV (Dingtong technology development co. LTD, Dalian, China), and the distance between the needle and collector was 12 cm. Thus, white mats consisting of interweaved PAN NFs were obtained on aluminum foil.

### 2.2. Fabrication of BiOBr/PAN Composite Mats (CMs)

Here, 0.75 mmol of Bi(NO_3_)_3_·5H_2_O and 0.75 mmol of cetyltrimethylammonium bromide (CTAB, Sinopharm Chemical ReagentCo., Ltd., Shanghai, China) were dissolved into a mixture solution consisting of 10 mL of ethylene glycol (Sinopharm Chemical ReagentCo., Ltd., Shanghai, China) and 30 mL of isopropyl alcohol (Sinopharm Chemical ReagentCo., Ltd., Shanghai, China). Then, 30 mg of the as-electrospun PAN NF mats were added to this solution under magnetic stirring for 3 h. Subsequently, the PAN mat-suspended mixture solution was transferred into a 50-mL Teflon-lined stainless-steel autoclave at 160 °C for 8 h. Then, the autoclave was cooled down to room temperature naturally. Finally, the obtained BiOBr/PAN CMs were removed from the autoclave, washed with deionized water and ethanol several times, and then dried in an oven at 60 °C for 8 h.

### 2.3. Fabrication of AgBr/BiOBr/PAN CMs

The AgBr/BiOBr/PAN CMs were prepared through an in situ ion exchange route, for which the as-fabricated BiOBr/PAN CMs were immersed into 20 mL of ethylene glycol with 2 mmol of AgNO_3_. The component ratio of the AgBr/BiOBr heterostructures on the electrospun PAN NFs was adjusted by controlling the reaction time at 2, 6, 10, and 12 h. Then, the products were washed with deionized water and ethanol several times. Finally, the obtained samples were dried in an oven at 60 °C for 8 h. The AgBr/BiOBr/PAN CMs obtained through the ion exchange treatment at 2, 6, 10, and 12 h were denoted as AgBr/BiOBr/PAN CMs-1, AgBr/BiOBr/PAN CMs-2, AgBr/BiOBr/PAN CMs-3, and AgBr/BiOBr/PAN CMs-4, respectively.

### 2.4. Characterization

Field emission scanning electron microscopy (FE-SEM; SU70, Hitachi, Japan) and X-ray diffraction (XRD; D/max2600, Rigaku, Japan) were used to characterize the morphology and the crystal structure of the products. An energy-dispersive X-ray (EDX) spectroscope coupled to an FE-SEM (SU70, Hitachi, Tokyo, Japan) was used to analyze the composition of the samples. The UV-Vis diffuse reflectance spectra of the samples were recorded on a Cary 500 UV-vis-NIR spectrophotometer (Agilent, Beijing, China).

### 2.5. Photocatalytic Tests

Here, 100 mg of the as-fabricated samples were put into 50 mL of methyl orange (MO, Sinopharm Chemical ReagentCo., Ltd., Shanghai, China) solution with an initial concentration of 10 mg/L, which was irradiated with a 300-W Xe lamp (Prefectlight, Beijing, China) equipped with an ultraviolet cut-off filter to provide visible light at λ ≥ 400 nm. The catalytic reaction was conducted at room temperature. Decreases in the concentrations of the dyes were analyzed by a Cary 500 UV-vis-NIR spectrophotometer at 460 nm. During the photocatalytic process, 3 mL of the MO solution was extracted at an interval of 15 min, and the samples were analyzed.

## 3. Results and Discussion

**Figure 1:**
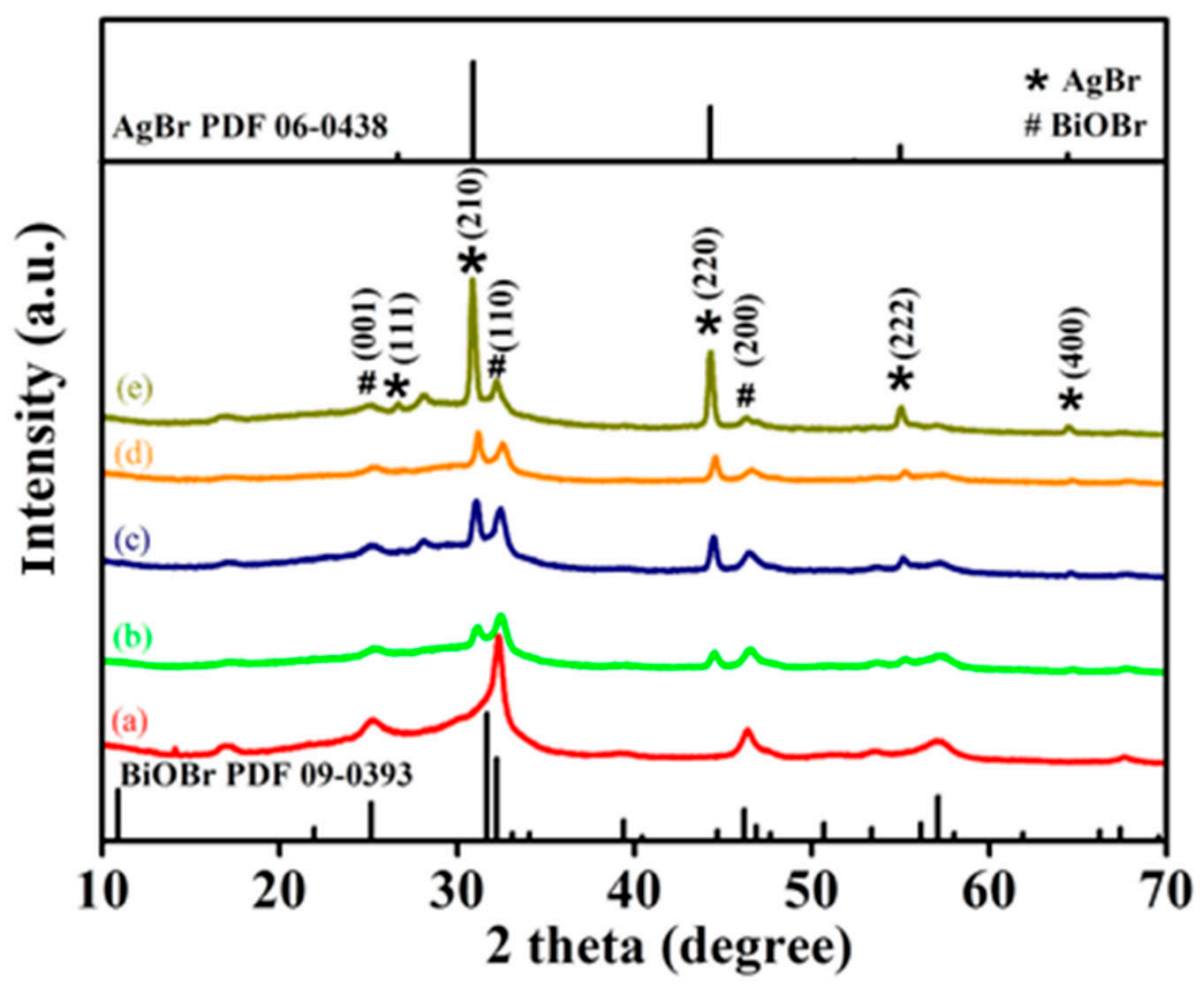
X-ray diffraction (XRD) patterns of the as-fabricated samples: (**a**) BiOBr/PAN CMs; (**b**) AgBr/BiOBr/PAN CMs-1; (**c**) AgBr/BiOBr/PAN CMs-2; (**d**) AgBr/BiOBr/PAN CMs-3; (**e**) AgBr/BiOBr/PAN CMs-4.

The phase structures of the as-fabricated samples were identified through X-ray diffraction (XRD) patterns. As observed in Figure 1, the sample of PAN NF mats only showed a very broad diffraction peak at 25° due to the semicrystalline structure of the polymer [16]. The diffraction peaks from the curve of the BiOBr/PAN CMs were indexed for tetragonal BiOBr (JCPDS, 09-0393). This indicated the formation of BiOBr nanostructures on the electrospun PAN NFs through the solvothermal process. After an ion exchange treatment, some new diffraction peaks originating from the AgBr (JCPDS, 06-0438) could be found in the XRD patterns of the AgBr/BiOBr/PAN CMs. Meanwhile, when we prolonged the reaction time during the ion exchange process, the diffraction intensities of the AgBr peaks were increased on the XRD curve of the AgBr/BiOBr/PAN CMs. This suggests that the component ratio between the AgBr and BiOBr in the ternary CMs could be adjusted by controlling the reaction time of the ion exchange process.

**Figure 2:**
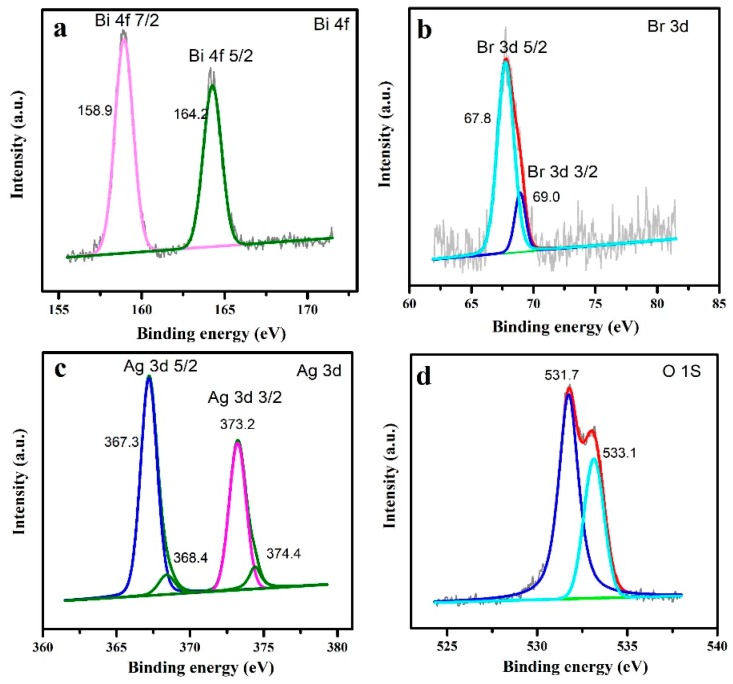
X-ray photoelectron spectroscopy (XPS) spectra of the AgBr/BiOBr/PAN CMs-2: (**a**) Bi 4f; (**b**) Br 3d; (**c**) Ag 3d; and (**d**) O 1s.

More detailed information regarding the chemical and bonding environment of the AgBr/BiOBr/PAN photocatalysis was ascertained using X-ray photoelectron spectroscopy (XPS). As shown in Figure 2a, two peaks at 158.9 and 164.2 eV were attributed to Bi 4f_7/2_ and Bi 4f_5/2_, respectively, which was indicative of Bi^3+^ in BiOBr. The Br 3d_5/2_ and Br 3d_3/2_ peaks were associated with binding energies of 67.8 and 69.0 eV, respectively (Figure 2b). The peaks at 367.3 and 373.2 eV were attributed to Ag^+^ in AgBr, and those at 368.4 and 374.4 eV were assigned to Ag^0^ species (Figure 2c). The O 1s peak could be fitted by two peaks at 531.7 and 533.1 eV, which were related to the oxygen in the BiOBr catalyst and other components (such as –OH and H_2_O) adsorbed on the surface of AgBr/BiOBr, respectively (Figure 2d).

**Figure 3:**
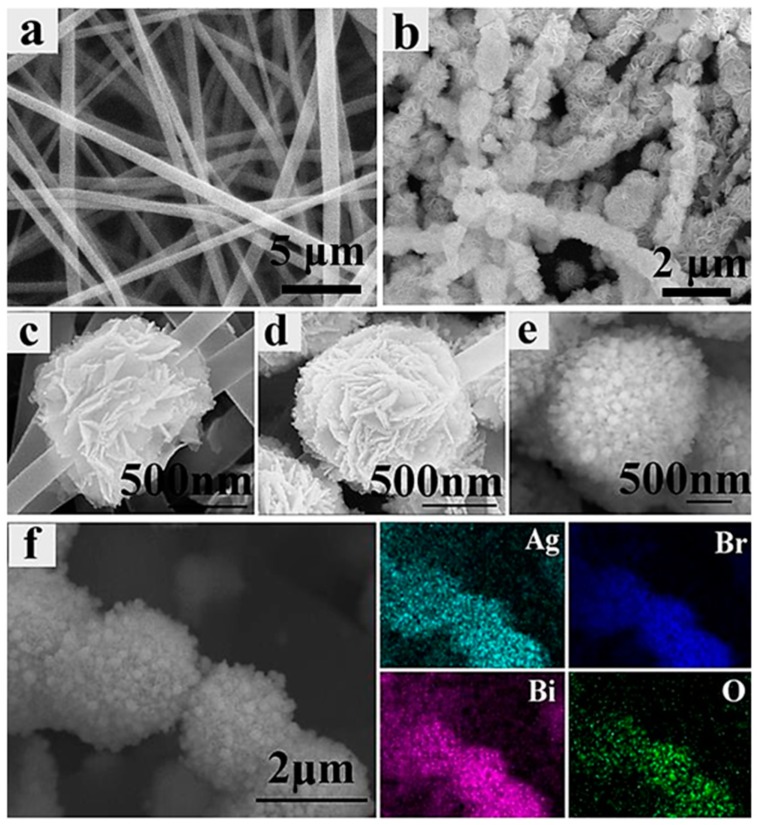
Scanning electron microscopy (SEM) images of (**a**) PAN nanofibers (NFs) and (**b**) BiOBr/PAN CMs; SEM images of BiOBr or AgBr/BiOBr nanostructures in (**c**) BiOBr/PAN CMs, (**d**) AgBr/BiOBr/PAN CMs-1, and (**e**) AgBr/BiOBr/PAN CMs-2; (**f**) SEM images of AgBr/BiOBr/PAN CMs-2 (left) and the corresponding elemental maps (right) of Ag, Br, Bi, and O.

The morphologies of the PAN NF mats, the BiOBr/PAN CMs, and the different AgBr/BiOBr/PAN CMs were characterized using scanning electron microscopy (SEM). Figure 3a shows that the electrospun PAN NFs had relatively smooth surfaces due to organic semicrystalline properties. After solvothermal treatment, BiOBr nanostructures could be observed on the surfaces of the electrospun PAN NFs (Figure 3b). The obtained BiOBr/PAN composite NFs remained as one-dimensional (1D) fibrous structures. It can be clearly seen that the flower-like BiOBr nanostructure on the PAN NF consisted of several standing nanosheets with thicknesses of 5–15 nm (Figure 3c). When the BiOBr/PAN CMs were immersed into the ethylene glycol solution for ion exchange with the Ag^+^ ions, AgBr NPs were produced in situ on the surfaces of the BiOBr nanosheets (Figure 3d,e). During this process, BiOBr nanosheets on the PAN NF surfaces provided reaction sites for AgBr loading. With an increase in the ion exchange time from 2 to 6 h, the amounts and sizes of the AgBr NFs on the BiOBr nanosheets significantly increased from AgBr/BiOBr/PAN CMs-1 to AgBr/BiOBr/PAN CMs-2 (Figure 3d,e). An SEM image of an individual AgBr/BiOBr/PAN NF and the corresponding energy-dispersive X-ray (EDX) map are shown in Figure 3f. Observation of the Ag-related image confirmed that the Ag atoms were indeed exchanged with the BiO^+^ to form AgBr NPs on the BiOBr nanostructures. Meanwhile, the Ag, Br, Bi, and O atoms were distributed uniformly in the AgBr/BiOBr/PAN NFs. The above results indicate that the AgBr NPs were successfully immobilized on the surfaces of the BiOBr nanosheets with good dispersion and a controllable density.

**Figure 4:**
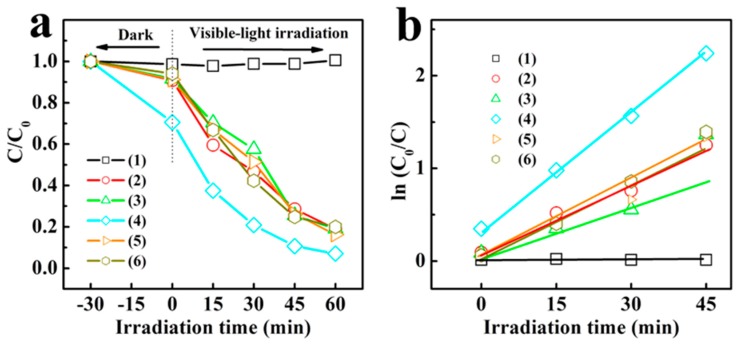
(**a**) Photocatalytic degradation curves and (**b**) the corresponding kinetic linear simulation curves of methyl orange (MO) over the different samples: (1) PAN nanofiber; (2) BiOBr/PAN CMs; (3) AgBr/BiOBr/PAN CMs-1; (4) AgBr/BiOBr/PAN CMs-2; (5) AgBr/BiOBr/PAN CMs-3; (6) AgBr/BiOBr/PAN CMs-4. C_0_: the initial concentration of MO; C: the residual concentration of MO.

**Scheme 2:**
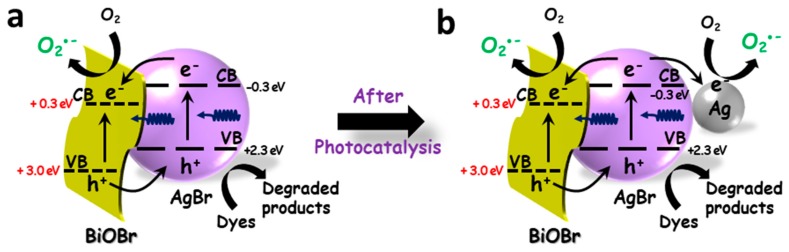
Schematic diagram showing the energy band structure and charge–transfer process in the heterostructure photocatalyst before and after the photocatalysis.

The photocatalytic activities of the as-fabricated samples were evaluated through the degradation of MO in water under visible-light irradiation. The characteristic absorption peak of MO at around λ = 460 nm was used to monitor the photocatalytic degradation process. The degradation efficiency was defined as *C*/*C*_0_, where *C* and *C*_0_ are the remnant and initial concentrations of MO, respectively. Before the photocatalytic tests, all of the samples were stirred for 30 min to achieve saturation adsorption. The photodegradation efficiencies of MO mediated by the series of photocatalysts are given in Figure 4a. It was found that the PAN NF mats possessed a poor ability to adsorb MO. In addition, no photocatalytic degradation of MO was observed on the PAN NF mats under visible-light irradiation due to the lack of semiconductor photocatalysts. When loading the BiOBr nanostructures on the PAN NF mats to form the BiOBr/PAN CMs, the photocatalytic activity of the MO degradation increased dramatically. After visible-light irradiation for 60 min, the photodegradation efficiency reached 81.0% over the BiOBr/PAN CMs. Notably, the AgBr/BiOBr/PAN CMs showed higher photocatalytic activities than the BiOBr/PAN CMs did for the degradation of MO under visible-light irradiation. Meanwhile, the photocatalytic activity of the AgBr/BiOBr/PAN CMs for the MO degradation was dependent on the ion exchange time during the synthesis process for the AgBr/BiOBr/PAN CMs. This observation indicates that the AgBr/BiOBr heterojunction played an important role in the enhancement of photocatalytic activity on the ternary CMs due to an effective interfacial charge–transfer (Scheme 2a). The optimal sample of AgBr/BiOBr/PAN CMs-2 could degrade 92.9% of MO under visible-light irradiation for 60 min. This photocatalytic activity was obviously higher than the reported flexible photocatalysts due to the existence of a semiconductor heterojunction to boost the separation of photoinduced charge carriers (Table 1). For a better understanding of the photocatalytic activity of the above samples, kinetic analyses of the degradation of MO are given in Figure 4b according to a pseudo-first-order reaction model: ln(*C_0_*/*C*) = *K_app_t*, where *K_app_* is the apparent first-order rate constant (min^−1^), and *t* is the time for visible-light irradiation. The linearity between ln(*C_0_*/*C*) and irradiation time was good for all of the photocatalysts, indicating that the photocatalytic degradation of MO in water could be described by pseudo-first-order reaction dynamics. The apparent rate constant of AgBr/BiOBr/PAN CMs-2 was 0.0511 min^−1^.

**Figure 5:**
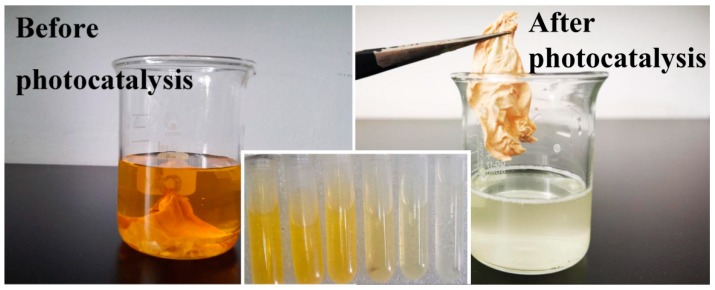
An optical image of macroscopical AgBr/BiOBr/PAN CMs removed from the photocatalytic reaction solution after photodegradation.

**Figure 6:**
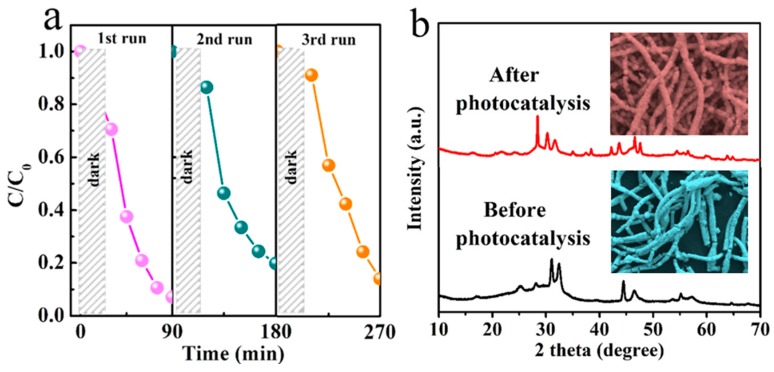
(**a**) Cycling test curves of the AgBr/BiOBr/PAN CMs-2 for the photocatalytic degradation of MO; (**b**) XRD patterns and SEM images of AgBr/BiOBr/PAN CMs-2 before and after three photocatalytic reactions.

In addition to the photocatalytic efficiency, the recyclability and stability of the photocatalyst are also crucial issues for practical applications. As shown in Figure 5, AgBr/BiOBr/PAN CMs-2 could be easily separated and recovered from solution after the photocatalytic reaction because of the macrostructural flexible structure of the electrospun fibrous mats [31,32,33]. After three cycles, the photocatalytic activity of AgBr/BiOBr/PAN CMs-2 was almost unchanged in terms of MO degradation (Figure 6a). This suggests that the as-fabricated photocatalyst possessed both recycling properties and stable photocatalytic activity.

The morphologies and crystalline structures of AgBr/BiOBr/PAN CMs-2 after three cycles were studied in comparison to AgBr/BiOBr/PAN CMs-2 before use in a photocatalytic reaction. As shown in Figure 6b, the photocatalyst of AgBr/BiOBr/PAN CMs-2 still had a 1D structure with secondary nanostructure loading. In addition, some NPs appeared on the surface of AgBr/BiOBr/PAN CMs-2, which could be attributed to the formation of a small amount of Ag NPs due to the photocatalytic reduction process.

Meanwhile, the intensities and positions of the diffraction peaks in the XRD patterns of AgBr/BiOBr/PAN CMs-2 were changed slightly before and after three cycles of photocatalytic reaction (Figure 6b). There were some new diffraction peaks in the XRD patterns of the AgBr/BiOBr/PAN CMs-2 used, which was in accordance with cubic Ag. This further confirmed the formation of Ag NPs on the AgBr/BiOBr/PAN CMs-2 used. Please note that the Ag NPs on the surface of the AgBr/BiOBr heterostructures could also boost the separation of photoinduced charge carriers by enhancing photocatalytic activity due to the “electron sink” effect of Ag NPs (Scheme 2b). Further investigation found that no detachment of AgBr/BiOBr nanostructures was observed during the photocatalytic reaction, indicating that the photocatalyst of AgBr/BiOBr/PAN CMs-2 had excellent photoactivity and good stability.

## 4. Conclusions

In summary, the AgBr/BiOBr hetero-nanostructures were controlled and immobilized on the flexible support of electrospun PAN NFs through a facile solvothermal process combined with an in situ ion exchange method. The AgBr/BiOBr/PAN CMs exhibited enhanced photocatalytic activity in the decomposition of MO compared to the BiOBr/PAN CMs. The enhanced photocatalytic activity could be attributed to extended absorption in the visible light region and the effective separation of photogenerated charge carriers across the AgBr/BiOBr heterojunction interface. Moreover, the AgBr/BiOBr/PAN CMs could be reclaimed easily without a decrease in the initial photocatalytic activity due to the processible flexibility induced by their unique hierarchical inorganic/organic heterostructure. It is believed that our present work offers a new platform to develop semiconductor heterojunction-based flexible photocatalysts for practical applications in water purification.

## Figures and Tables

**Table 1:** The photocatalytic degradation parameters over the different nanofiber photocatalysts.

Photocatalyst	Light	Photocatalytic Results	Year	Ref.
AgI–BiOI/PAN composite nanofibers	300-W Xe lamp (λ > 400 nm)	The photodegradation efficiency of Rhodamine B (RhB) could reach 98.5% within 210 min	2018	[14]
BiOCl/CuTNPc/PAN nanofibers	150-W xenon lamp (λ > 420 nm)	The degradation rate of RhB reached 75% within 180 min	2018	[15]
CuAl_2_O_4_ hollow nanofibers	500-W xenon lamp (λ ≧ 420 nm)	The RhB and MO solutions were degraded by 83.5% and 94% within 360 and 300 min	2018	[16]
Polyoxometalate/TiO_2_/Ag composite nanofibers	300-W Xe lamp (λ ≧ 420 nm)	MO (20 mL, 20 ppm) could be completely degraded within 160 min	2018	[17]
PAN/g-C_3_N_4_/BiOI nanofibers	500-W Xe lamp (λ ≧ 400 nm)	The degradation efficiency of RhB could reach 98.0% after irradiation for 90 min	2018	[18]
g-C_3_N_4_ nanosheets/TiO_2_ nanofibers	500-W xenon lamp (λ ≧ 420 nm)	The photocatalytic degradation ratios of RhB could reach 93.6% after irradiation for 120 min	2018	[19]
p-BiOCl/nZnFe_2_O_4_ nanofibers	150-W xenon lamp (λ > 420 nm)	The photodegradation efficiency of RhB could reach 97% within 180 min	2018	[20]
ZnO/γ-Al_2_O_3_ nanofibers	120-W ultraviolet radiation lamp	The MO could be completely degraded in 120 min	2018	[21]
Ag nanowire@carbon nanofiber@Ag nanoparticles	300-W Xe lamp (λ > 400 nm)	91% of methylene blue (MB) could be eliminated within 120 min	2018	[22]
CuO nanofibers		The degradation rate of RhB reached 96% within 160 min	2018	[23]
Bi_2_MoO_6_/NiTiO_3_ nanofibers	300-W Xe lamp (λ ≧ 420 nm)	The degradation efficiency of MB, MO, and RhB achieved using 1.0 Bi–Ni was 100%, 86.7%, or 100% within 90 min	2018	[24]
Bi_2_WO_6_/ZnFe_2_O_4_ nanofibers	150-W xenon lamp (λ > 420 nm)	The degradation rate of RhB reached 98% within 300 min	2018	[25]
ZnIn_2_S_4_/TiO_2_ nanofibers	350-W Xe lamp (λ ≧ 420 nm)	The MO could be completely degraded within 75 min	2018	[26]
Bi_2_MoO_6_/BiFeO_3_ heterojunction nanofibers	150-W xenon lamp (λ > 420 nm)	The degradation rate of RhB reached 98% within 5 h	2018	[27]
CuCrO_2_/SnO_2_ nanofibers	250-W metal halide lamp	The MB could be completely degraded within 120 min	2018	[28]
Ag_2_O/TiO_2_ composite nanofibers	visible light irradiation (>420 nm)	The degradation rate of RhB reached 87.7% within 80 min	2019	[29]
Au/BiFeO_3_ composite nanofibers	500W xenon lamp	The degradation rate of RhB reached 85.76% within 3h	2019	[30]
